# Nitrogen Incorporated Photoactive Brownmillerite Ca_2_Fe_2_O_5_ for Energy and Environmental Applications

**DOI:** 10.1038/s41598-020-59454-w

**Published:** 2020-02-17

**Authors:** Durga Sankar Vavilapalli, Soma Banik, Raja Gopal Peri, Muthuraaman B., Muralidhar Miryala, Masato Murakami, Klimkowicz Alicja, Asokan K., Ramachandra Rao M. S., Shubra Singh

**Affiliations:** 1 Crystal Growth Centre, Anna University, Chennai, 600025 India; 2 Synchrotron Utilization Section, Raja Ramanna Centre for Advanced Technology, Indore, 452013 India; 3 Homi Bhabha National Institute, Training School Complex, Anushakti Nagar, Mumbai 400094 India; 4 Department of Energy, University of Madras, Chennai, 600025 India; 5 Graduate School of Science and Engineering, Shibaura Institute of Technology, 3-7-5 Toyosu, Koto-ku, Tokyo 135-8548 Japan; 6 Materials Science Division, Inter University Accelerator Centre, New Delhi, 110067 India; 7 Nano Functional Materials Technology Centre, Department of Physics, Indian Institute of Technology Madras, Chennai, 600036 India

**Keywords:** Energy science and technology, Materials science, Nanoscience and technology, Physics

## Abstract

Ca_2_Fe_2_O_5_ (CFO) is a potentially viable material for alternate energy applications. Incorporation of nitrogen in Ca_2_Fe_2_O_5_ (CFO-N) lattice modifies the optical and electronic properties to its advantage. Here, the electronic band structures of CFO and CFO-N were probed using Ultraviolet photoelectron spectroscopy (UPS) and UV-Visible spectroscopy. The optical bandgap of CFO reduces from 2.21 eV to 2.07 eV on post N incorporation along with a clear shift in the valence band of CFO indicating the occupation of N 2p levels over O 2p in the valence band. Similar effect is also observed in the bandgap of CFO, which is tailored upto 1.43 eV by N^+^ ion implantation. The theoretical bandgaps of CFO and CFO-N were also determined by using the Density functional theory (DFT) calculations. The photoactivity of these CFO and CFO-N was explored by organic effluent degradation under sunlight. The feasibility of utilizing CFO and CFO-N samples for energy storage applications were also investigated through specific capacitance measurements. The specific capacitance of CFO is found to increase to 224.67 Fg^−1^ upon N incorporation. CFO-N is thus found to exhibit superior optical, catalytic as well as supercapacitor properties over CFO expanding the scope of brownmillerites in energy and environmental applications.

## Introduction

Multifunctional brownmillerite Ca_2_Fe_2_O_5_ is a promising material for energy and environmental applications such as fuel cells, supercapacitors, batteries, H_2_ production and CO_2_ capture, attributed mostly to its multifaceted property like those in catalysis and mixed ionic electronic conduction (MIEC)^1–5^. Presence of a visible region bandgap along with its catalytic activity also enables it as a photoactive material and most importantly as material for textile waste water remediation. There is a huge need for industrial waste water purification of the effluents from the textile industries before releasing it to water bodies. A lower cost and energy requirement pushes us to explore more efficient materials which can absorb a larger percentage of incident natural sunlight and make their impact felt on the environment^6–10^. Well known wide band gap semiconductors, such as TiO_2_ and ZnO (bandgap > 3 eV), cannot perfectly match the broad ranges of solar radiation emphasizing the need to investigate new materials/composites with narrow bandgap^11^. Quite recently perovskite metal oxides, such as PbTiO_3_ (2.75 eV), AgNbO_3_ (2.86 eV), SrNbO_3_ (1.9 eV), BiFeO_3_ (2.1 eV), LaFeO_3_ (2.4 eV), LaNiO_3_ (2.42 eV) have been found to possess reasonable catalytic efficiency^12–19^. This encourages us to work with novel materials like oxygen deficient perovskites for sunlight-driven photocatalysis.

To meet the above objectives, it is desirable to modify such structures with transition metal-N_x_ active sites to enhance the charge transport features and hence the catalytic activity towards remediation of industrial wastewater^20,21^. Recently, Nitrogen-doped layered perovskite K_2_La_2_Ti_3_O_10_ was shown to exhibit enhanced photocatalytic activity over Degussa P25 while degrading organic effluents under visible light^22^. Both LaNiO_3_ and double perovskite-structured rGO-NdBa_0.5_Sr_0.5_Co_1.5_Fe_0.5_O_5+δ_ have also shown enhanced catalytic performance with N-doping^23^.

An efficient way to improve photocatalytic performance lies in reducing the electron-hole recombination^24^. Oxygen defects present in the photocatalysts act as photoinduced charge traps providing adsorption sites and transferring the charge to the adsorbed compounds, thus preventing the recombination of photogenerated charge carriers. The process results in an overall improvement of the photocatalytic performance. A perfect example is that of oxygen deficient perovskite showing an enhanced catalytic efficiency for H_2_ production as well as photocatalytic activity^25–27^. This makes it evident that the catalyst system incorporating the advantageous features of oxides with substantial oxygen vacancies and transition metal-N interaction in a single stable structure will have desirable enhanced catalytic activity. Ca_2_Fe_2_O_5_ (CFO) possesses a significant combination of oxygen vacancies as well as a narrow bandgap ~1.8 to 2.2 eV (falling under visible region of solar spectrum). Both perovskites and brownmillerites exhibit photocatalytic activity due to the existence of active FeO_6_ octahedra in the crystal structure. CFO composites and metal-supported brownmillerites have already been used for water-splitting and as well as for oxidizing CO^28,29^. Though these reports tend to reveal the photodegrading ability of CFO, the photodegradation rate is much slower. An efficient way to enhance the photocatalytic performance of brownmillerite CFO is by incorporating N for efficient wastewater treatment. Since CFO is a mixed ionic-electronic conductor (MIEC), it has a strong potential to be used for supercapacitor applications^2,30,31^. N incorporation in metal-oxides can enhance the electrical conductivity, corrosion resistance as well as stabilty. Some of these properties are desirable for supercapacitor applications^32,33^. Detailed investigation on the feasibility of using nitrogen incorporated CFO (CFO-N) for solar energy utilization as well supercapacitor applications is the focus of this work.

## Experimental

Polycrystalline single phase Ca_2_Fe_2_O_5_ was synthesized by chemical route^5^. High pure chemicals of Ca, Fe nitrates were dissolved in water and proportionate amount of citric acid (CA) was added and stirred at room temperature. Ammonia solution was then added to maintain the pH ~ 6–7 and temperature of hot plate was increased to 80 °C. Once the solution turns viscous, ethylene glycol (EG) was added to the suspension to maintain the ratio of EG/CA as 1.2. The product was then heated at 300 °C to obtain a dry mass which was calcined at 700 °C for 6 h in box furnace to obtain nanostructured CFO. To enable N incorporation, as synthesized CFO was heated at 700 °C for 6 h in NH_3_ gas flow with a constant flow rate. The as-prepared powder sample was calcined at 500 °C for 4 h to avoid surface absorbed OH species and to obtain single phase nitrogen incorporated CFO (CFO-N).

X-ray powder diffraction (XRD) of these samples were performed by a Bruker D2 X-ray diffractometer using Cu Kα radiation. The microstructure and elemental mapping of as-synthesized samples were recorded using scanning electron microscope (SEM, Jeol, 20KV) and Energy-dispersive X-ray spectroscopy (EDX) respectively. The High-resolution transmission electron microscopy (HRTEM) images were recorded using a field emission transmission electron microscope (JEOL) at an accelerating voltage of 200 kV. The UV–visible absorption spectra of the samples were recorded on a UV–visible spectroscope (Jasco V-730). The chemical states present in CFO and CFO-N samples were analyzed using X-ray photoemission spectroscopy (XPS) with Al Kα source from SPECS (XR 50) at the experimental station of angle-resolved photoelectron spectroscopy (ARPES), Indus-2, India. To obtain atomically clean surface, the samples were sputtered with Argon ions at 1 keV for 10 min. C 1s (284.6 eV) was used to calibrate the peak position of the core levels. In order to investigate the valence band of CFO and CFO-N, the excitation energy of monochromatic He-1 line from SPECS (UVS 300) was used. The photoemission data was recorded with a SPECS Phoibos 150 electron energy analyzer and the base vacuum during the measurement was 7 × 10^−11^ mbar. Cyclic voltammetry and charge-discharge studies were investigated using Electrochemical workstation using Autolab (PGSTAT 204 FRA32M).

## Results and Discussions

**Figure 1:**
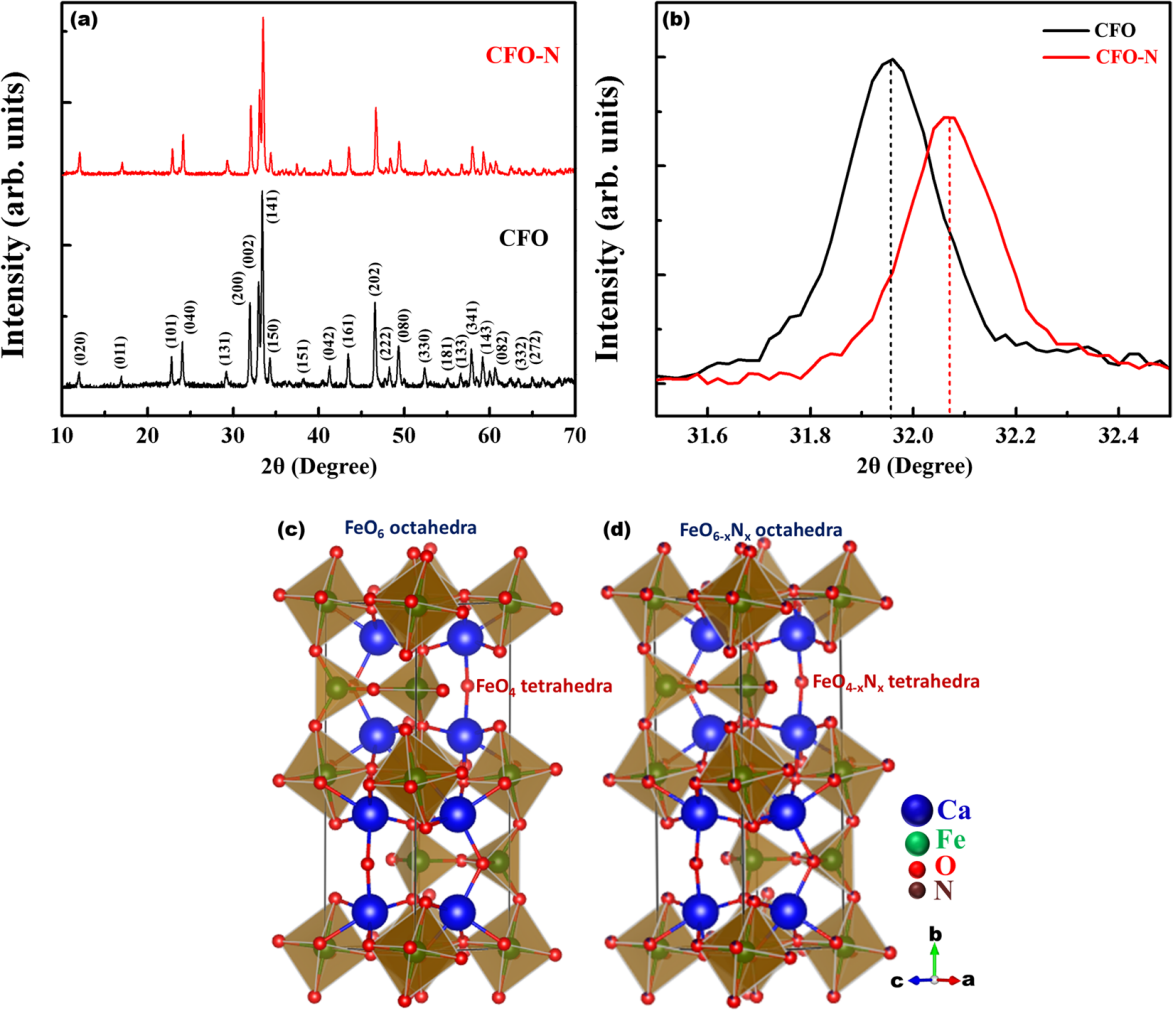
(**a**) XRD patterns for CFO and CFO-N (**b**) Magnified view of (200) plane in XRD showing a shift towards higher 2θ (**c**) Crystal structure of CFO with alternative layers of FeO_6_ octahedra and FeO_4_ tetrahedra (**d**) Crystal structure of CFO-N with partial incorporation of N in O sites.

X-ray diffraction patterns of both CFO and CFO-N are shown in figure 1a are in good agreement with the orthorhombic crystal system. Figure 1b shows a shift in peak position for (200) in CFO-N towards higher 2θ values implying the N incorporation^34,35^. Figures 1(c,d) show the crystal structure of CFO and CFO-N. The oxygen sites of FeO_6_ octahedra and FeO_4_ tetrahedra are partially replaced with N atoms, leading to the formation of FeO_6−x_N_x_ and FeO_4−x_N_x_ in CFO-N.

**Figure 2:**
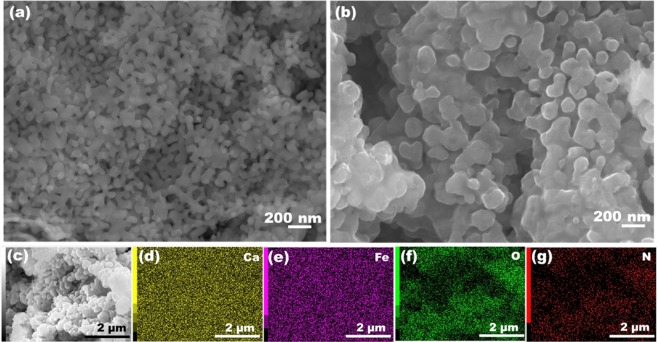
SEM images corresponding to (**a** and **b**) CFO and CFO-N (**c**–**g**) EDX mapping images of CFO-N sample showing uniform distribution of Ca, Fe, O and N elements.

**Figure 3:**
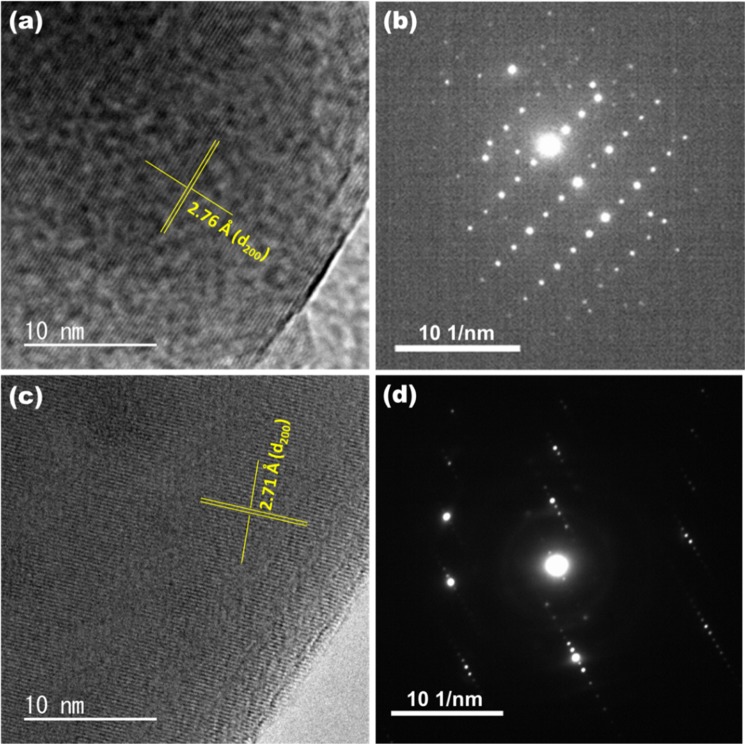
HRTEM and SAED images corresponding to (**a**,**b**) CFO and (**c**,**d**) CFO-N.

Figure 2a,b do not reveal any appreciable change in morphology upon N incorporation. EDX mapping of CFO-N (Fig. 2(c–g)) reveal the presence and uniform distribution of Ca, Fe, O and N elements throughout the compound. The EDX elemental mapping and spectra of CFO and CFO-N are shown in Figs. S1 and S2. EDX elemental mapping of CFO-N revealed that 3.36% atomic percentage of N was incorporated in CFO. Figure 3(a–d) show HRTEM and SAED micrographs of CFO and CFO-N samples having clear resolved crystalline domains corresponding to (200) lattice plane. The lattice spacing corresponding to (200) plane in CFO-N (2.71 Å) is found to be smaller than that of CFO (2.76 Å), which is also consistent with the small shift observed in XRD. The SAED pattern also establishes the crystalline nature of CFO and CFO-N samples.

**Figure 4:**
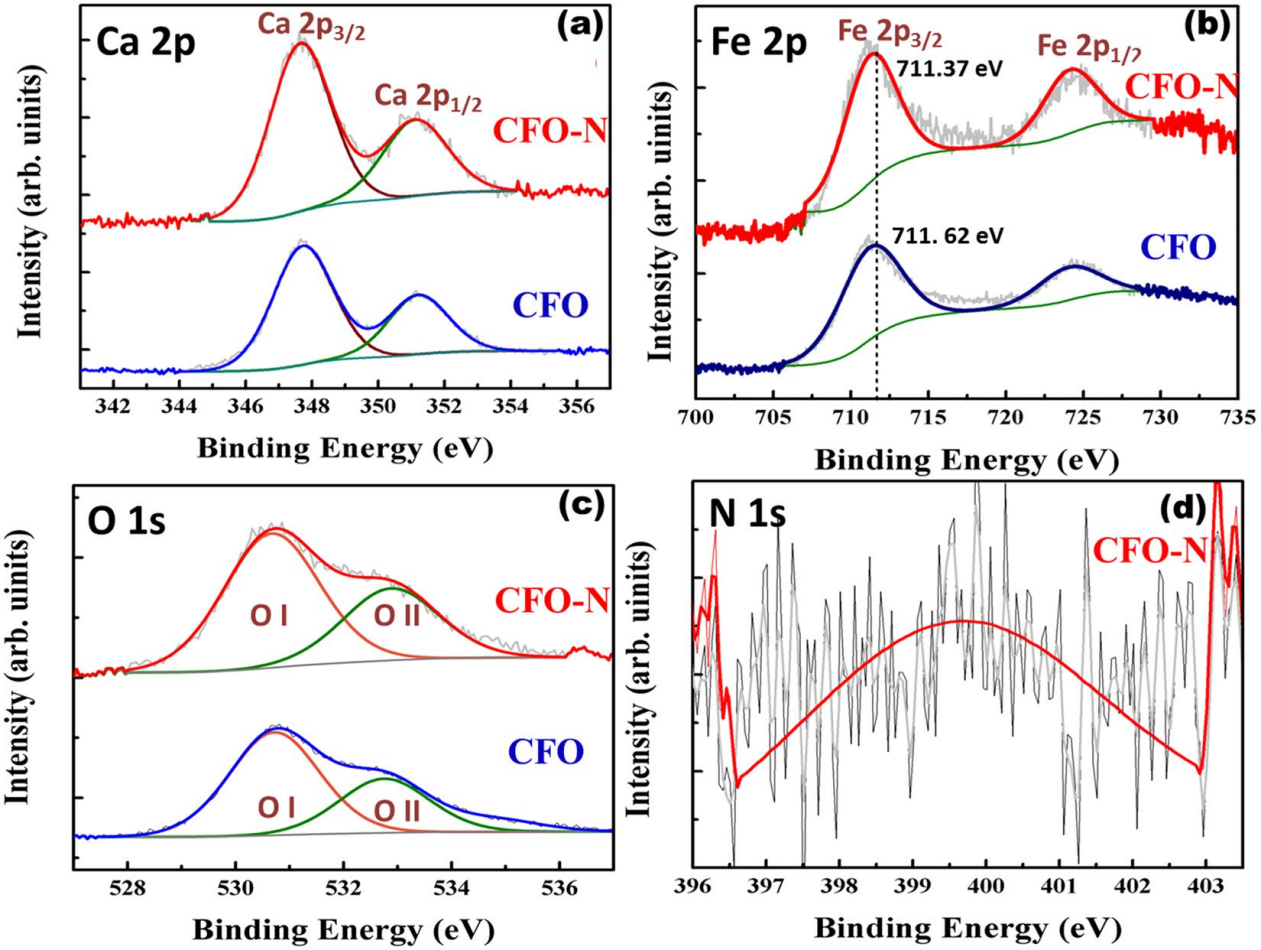
X-ray photoelectron spectra and the corresponding fits belonging to CFO and CFO-N samples: (**a**) Ca 2p, (**b**) Fe 2p, and (**c**) O 1s, (**d**) N 1s.

X-ray photoelectron spectroscopy (XPS) measurements of CFO and CFO-N confirm the valence states of constituent elements, simultaneously pointing towards the presence of nitrogen in CFO-N. XPS spectra corresponding to Ca 2p of CFO and CFO-N (Fig. 4(a)) reveal a split into doublet due to spin-orbit coupling of Ca 2p_3/2_ and Ca 2p_1/2_ with binding energy difference ~3.42 eV and ~3.41 eV respectively. The split points toward the existence of Ca in 2+ oxidation state in both these samples. Similarly for Fe, the Fe2p splits into two spin orbits Fe 2p_3/2_ and Fe 2p_1/2_ peaks (Fig. 4(b)). The positions of Fe 2p_3/2_ and Fe 2p_1/2_ spin orbits at 711.62 eV and 724.40 eV respectively for CFO confirms the existence of Fe in Fe^3+^ valence state^36^. A small shift in Fe 2p towards lower binding energy in CFO-N arises due to the replacement of O in Fe-O sites by N. Since N has lower electronegativity as compared to O, it leads to lower binding energy of Fe 2p in CFO-N by forming Fe-N_x_ sites^37^. The XPS spectra of O1s for CFO and CFO-N (Fig. 4(c)) were deconvoluted into two components at 530.72 eV (OI) and 532.75 eV (OII) for CFO and at 530.66 eV (OI) and 532.88 eV (OII) for CFO-N respectively. The OI component arises from the lattice oxygen, whereas OII is typical of hydroxyl and carbonate groups indicating possible chemisorption of oxygen species from the atmosphere^31^. The N 1s component appears at 399.5 eV (Fig. 4(d)) confirming the presence of nitrogen in CFO-N.

**Figure 5:**
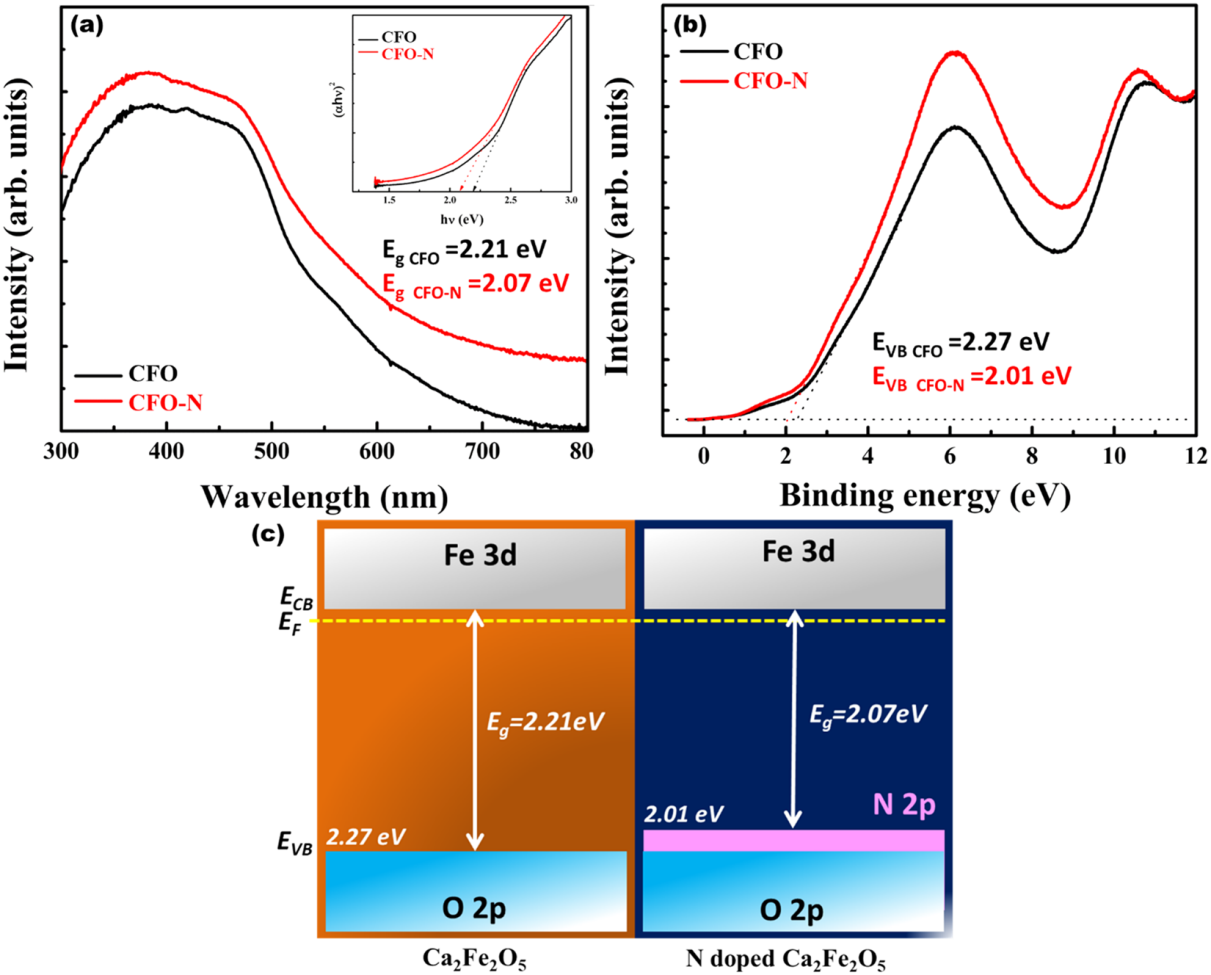
(**a**) UV-vis absorption spectra (inset shows corresponding Tauc plot) (**b**) Ultraviolet photoelectron spectra (**c**) Energy band diagram corresponding to CFO and CFO-N.

The electronic structures of CFO and CFO-N were probed by ultraviolet photoemission spectroscopy (UPS) measurements. It is a well-established fact that the valence band maximum (VBM) calculation can be performed by considering the cutoff for the lowest binding energy. A clear decrease in VBM is observed in CFO-N (2.01 eV) compared with CFO (2.27 eV) shown in Fig. 5b. This points towards occupation of N 2p levels over O 2p levels in valence band leading to an effective reduction in bandgap due to the lower electronegativity of N and creation of Fe-N_x_ active sites in CFO-N. Figure 5(a) illustrates the UV-visible absorption spectra corresponding to CFO and CFO-N. The absorption edge of CFO-N shifts towards higher wavelength indicating a significant reduction in bandgap for CFO-N (2.07 eV) over CFO (2.21 eV) (inset Fig. 5a). The corresponding energy band diagrams of CFO and CFO-N are shown in Fig. 5(c).

**Figure 6:**
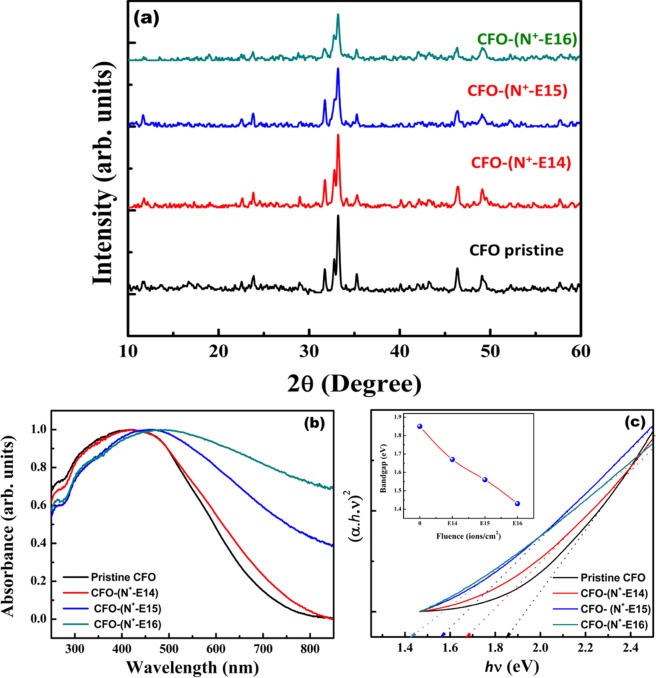
(**a**) XRD pattern of pristine CFO, N ion implanted CFO films with fluencies of 10^14^, 10^15^ and 10^16^ ions/cm^2^. (**b**) UV-Vis absorption spectra of pristine CFO, N ion implanted CFO films (**c**) corresponding Tauc plot to measure band gap (inset bandgap reduction plot by increasing fluence).

The bandgap of CFO could also be tailored by N^+^ ion implantation unlike conventional doping. In order to implant the N^+^ ions, Ca_2_Fe_2_O_5_ bulk films were fabricated on glass substrates using Doctor blade method. These CFO films were then implanted with 1 MeV of N^+^ ion beam with a beam current of 1 µA using low energy ion beam facility (LEIBF). The implantation was performed at three ion fluencies 10^14^, 10^15^ and 10^16^ ions per cm^2^, which were named as CFO-N^+-^-E14, CFO-N^+^-E15 and CFO-N^+^-E^16^ respectively. X-ray diffraction (XRD) measurements were performed to investigate the effect of N ion implantation on the crystal structure and the nature of crystallinity of CFO films. Figure 6(a) shows the XRD patterns of pristine CFO and N implanted CFO films. No additional diffraction peaks corresponding to other phases are detected.

Nitrogen (N) incorporation in CFO by implantation is also expected to change the optical properties by narrowing its bandgap due to reasons mentioned above. In the present case too, N^+^ ion implanted films showed modified optical properties as compared to pristine CFO (see Fig. 6(b)). Figure 6(c) shows the corresponding Tauc plot. The bandgap values were found to be 1.85, 1.67, 1.56 and 1.43 eV for pristine CFO, CFO-N^+^-E14, CFO-N^+^-E15 and CFO-N^+^-E16 respectively. These studies corroborate the effective reduction in bandgap of CFO due to N incorporation.

**Figure 7:**
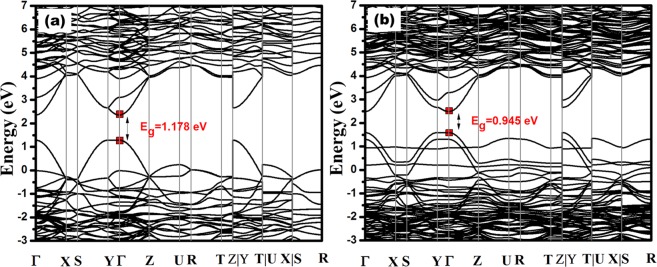
Computationally calculated band structures of (**a**) CFO and (**b**) CFO-N.

The electronic structures of CFO and CFO-N were also investigated theoretically by density functional theory (DFT) calculations, as shown in Fig. 7(a,b). These calculations were performed by sampling the Brillouin zone with a set of high symmetry k-points. The effect of Nitrogen incorporation in CFO was analyzed theoretically and the bandgap of CFO was found to be 1.17 eV. Upon replacement of few O atoms with N atoms in a unit cell of CFO, the bandgap reduces to 0.94 eV, strongly supporting the experimental trend. The computationally predicted bandgap values are lower than the experimental values. It is a well-known fact that density functional theory (DFT) calculations underestimate the actual values^38,39^ (Detailed computational procedure is discussed in supplementary information).

CFO and CFO-N have excellent optical and catalytic properties. To explore the use of CFO and CFO-N in environmental remediation applications, we have investigated sunlight driven photocatalytic degradation of organic effluent Mythylene blue (MB). The photodegradation experiments were carried out under natural sunlight at a latitude of 13.0087°N and longitude of 80.2371°E (Chennai, India). The initial dye (MB) concentration was kept at C_o_: 1 × 10^−5^ mol/L. Then 50 mg/100 ml catalyst loaded dye solution was ultrasonicated vigorously under the dark condition and allowed to rest in dark for 10 min to ensure good adsorption. The catalyst loaded dye solution was then kept in sunlight and the sample was collected at regular time intervals. Simultaneous light intensity measurements were also carried out using lux meter. For the whole duration of experiment (50 min) the incident intensity of sunlight was almost constant with an average intensity of about 105.40 klux. The degradation efficiency and first-order reaction kinetics are calculated using the following equations.

1 $$Percentage\,of\,Degradation\,({\rm{ \% }}D)=\left[\frac{{C}_{0}-C}{{C}_{0}}\right]\times 100$$

2 $$\mathrm{ln}\,\frac{{C}_{0}}{C}=kt$$

where C_o_ and C are the concentrations of MB at 0 min and at time interval *t* respectively. *k* is the degradation rate constant.

**Figure 8:**
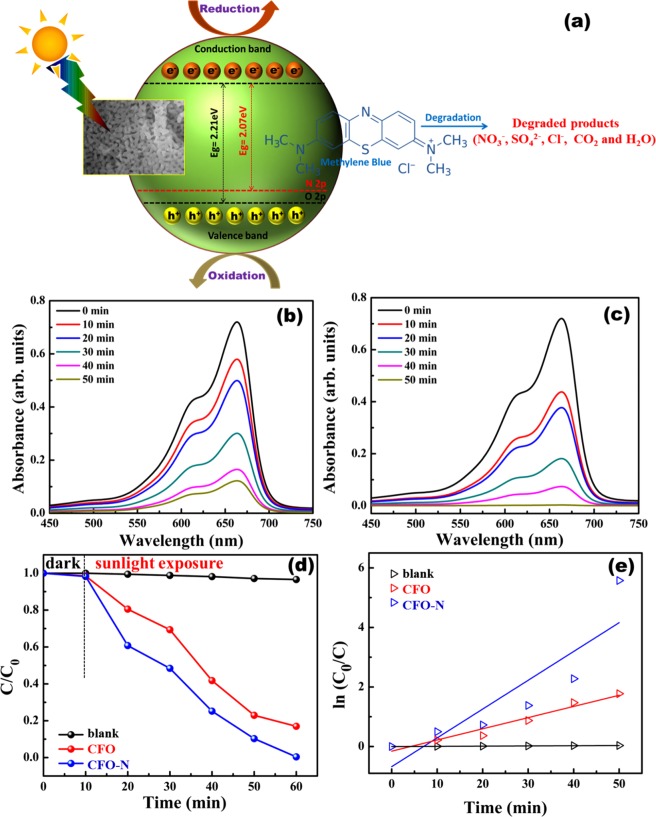
(**a**) Schematic representation of sunlight driven photodegradation mechanism of MB (**b**) degradation profile of MB by CFO (**c**) degradation profile of MB by CFO-N (**d**) C/Co for MB under natural sunlight (**e**) First order photocatalytic reaction kinetics.

The schematic representation of photodegradation process, along with degradation profile, efficiency and first-order reaction kinetics of CFO andCFO-N are shown in Fig. 8(a–e). The degradation efficiencies of bare MB, CFO and CFO-N are found to be 3.5%, 83% and 99.7% respectively and the first order rate constants of CFO and CFO-N are found to be 0.0375 min^−1^ and 0.096 min^−1^ respectively. CFO-N shows superior photocatalytic performance compared to CFO due to a relatively smaller bandgap as well as the presence of Fe-N_x_ active sites. Adsorption curves of MB and photodegradation performance CFO and CFO-N over different photocatalysts are discussed in supporting information.

**Figure 9:**
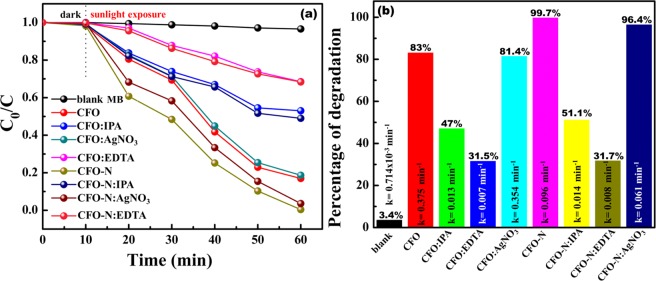
(**a**,**b**) Comparisons of photocatalytic activities of CFO and CFO-N for the degradation of MB with or without adding trapping agents.

The major active species involved in the photodegradation mechanism of MB was determined by the active species trapping experiments. The trapping experiment results are shown in Fig. 9(a,b). We have used various scavengers such as AgNO_3_ (1 mmol) as electron (e^−^) scavenger, Ethylenediaminetetraacetic acid (EDTA, 1 mmol) as a hole scavenger (h^+^) and Isopropyl alcohol (IPA, 1 mmol) as the OH^−^ radical trapping agent. The trapping agents were added to dye-catalyst solution and degradation experiments were performed under sunlight. In absence of trapping agents, the MB could be degraded by 83% and 99.7% of its initial concentration with the presence of CFO and CFO-N respectively. Upon addition of AgNO_3_ as an e^−^ scavenger to CFO and CFO-N dye solution the photodegradation efficiencies were found to be 81.4% and 96.4% respectively. No significant change in photodegradation performance leads us to infer that electrons (e^−^) are not involved in photodegradation mechanism of MB by both CFO and CFO-N. With the presence of EDTA as h^+^ scavenger photodegradation efficiencies of CFO and CFO-N were reduced to 31.5% and 31.7% respectively, confirming that photodegradation of MB using CFO and CFO-N are governed by holes. Reactive oxygen species (ROS) such as ^.^O_2_, ^·^OH, singlet oxygen (^1^O_2_), peroxyl (RO_2_^.^), and alkoxyl (RO^−^) are highly active in photochemical reactions such as photodegradation of organic pollutants^40^. Among the all ROS, superoxide (O_2_^.^) and hydroxyl(^·^OH) radicals are the most possible generated ROS in photodegradation of organic pollutants. In the present case, it is evident from e- and h^+^ trapping experiments, that photodegrading phenomena are governed by holes (h^+^) and there is no participation of e^−^ in photodegradation process. Hence, the generation of -O_2_^.^ species is limited. This is, because -O_2_^.^ can be generated upon reduction of O_2_ by e^−^ ^41^. In the present photochemical reaction, ^·^OH is one of the major ROS, which is generated by oxidation of H_2_O/OH^−^ by holes (h+). Since ^·^OH has high tendency to degrade organic pollutants^42^, ·OH species trapping experiment was conducted by addition of IPA; as a result of which the photodegradation efficiencies of CFO and CFO-N were reduced to 47% and 51.1% respectively. Hence, the active species trapping experiments suggest that, photodegradations of MB by CFO and CFO-N are attributed to the holes(h^+^) and ^·^OH active species.

**Figure 10:**
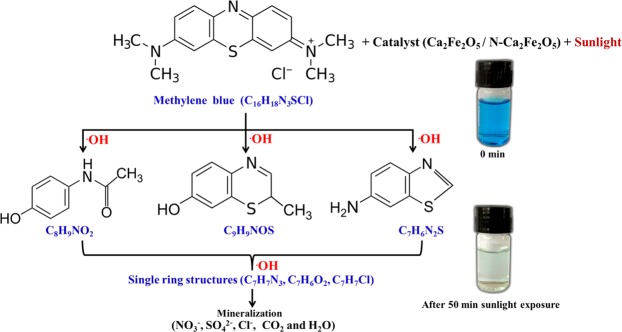
Possible photodegration pathway of MB in the presence of catalyst.

Figure 10 shows a possible MB photodegradation pathway in the presence of catalyst. During the photocatalytic degradation process, active species such as holes(h^+^) and hydroxyl radicals (^·^OH) are generated which are responsible for mineralization of MB into unstable organic byproducts Cl^−^, NO_3_^−^, SO_4_^2−^, CO_2_, and H_2_O^36,43^. MB degradation process is initiated by active species breaking the S-Cl bonds and then Cl^−^ ions being separated from MB core structure. Several intermediate molecules such as C_8_H_9_NO_2_, C_9_H_9_NOS and C_7_H_6_N_2_S are generated by breaking N-CH_3_ bonds, (which are connected to terminal of MB core structure). The active species further oxidizes the –CH_3_ groups. It also gives rise to single ring structures (C_7_H_7_N_3_, C_7_H_6_O_2_ and C_7_H_7_Cl). These reactions continue until the MB mineralizes into Cl^−^, NO_3_^−^, SO4^2−^, CO_2_, and H_2_O.

Ca_2_Fe_2_O_5_, by virtue of its unique properties as mentioned above, could also be used for energy harvesting applications such a supercapacitors, batteries and fuel cells etc. In this regard,we have explored the feasibility of CFO and CFO-N for supercapacitor applications. Electrochemical studies have been performed on CFO and CFO-N using three electrode system with 0.5 M Na_2_SO_4_ aqueous solution as the electrolyte. These techniques are employed for investigating the redox behavior, stability and supercapacitor properties. The working electrodes are prepared on FTO plates with ethanol as solvent using a doctor blade method.

**Figure 11:**
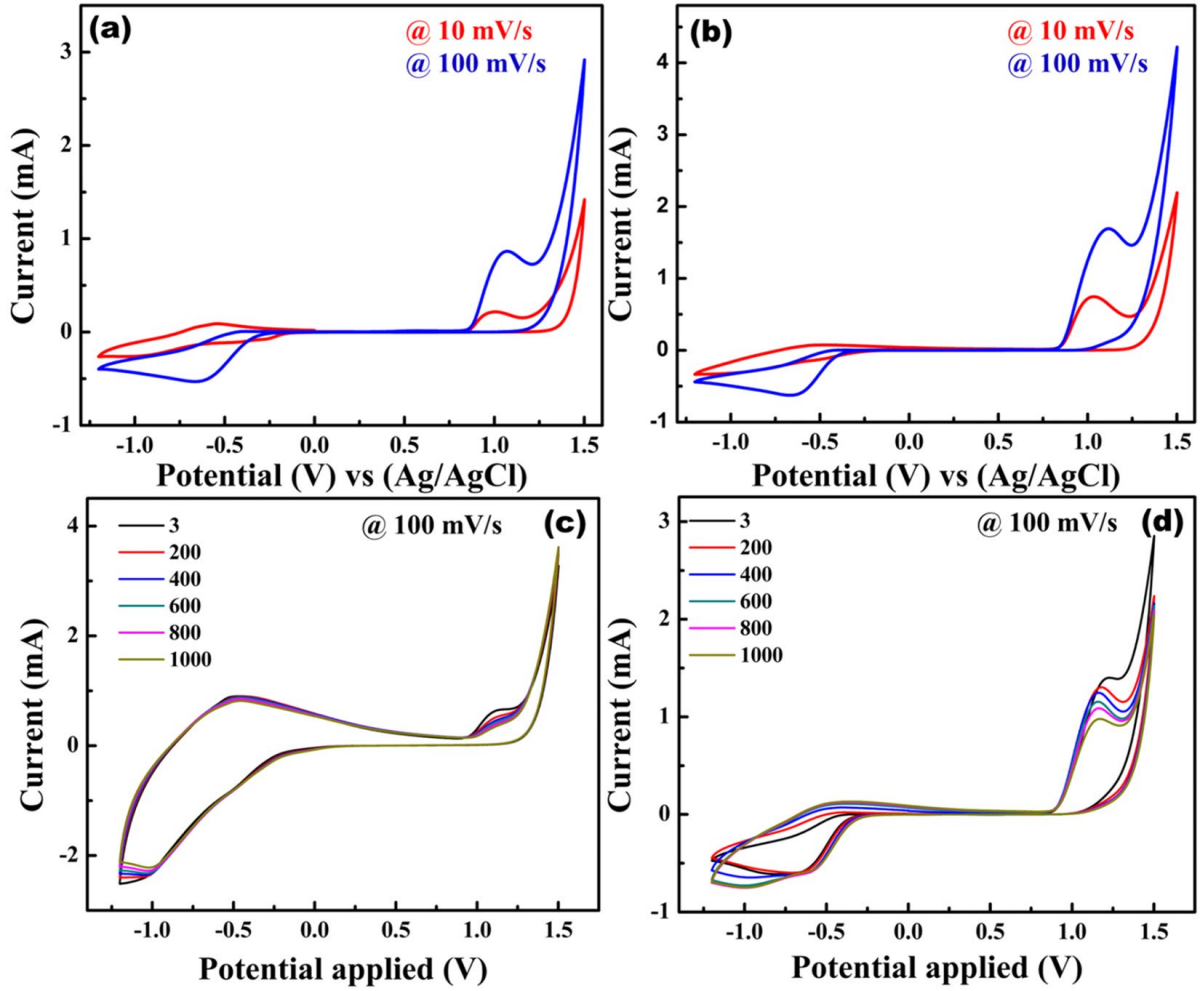
Cyclic voltammetric curves of (**a**) CFO at high and low scan rates 10 mV/s and 100 mV/s (**b**) CFO-N at high and low scan rates 10 mV/s and 100 mV/s (**c**,**d**) Cyclic stability of CFO for 1000 cycles (**d**) Cyclic stability of CFO-N for 1000 cycles.

Figure 11(a,b) shows the cyclic voltammetry (CV) curves of CFO and CFO-N at different scan rates (10 mV/s and 100 mV/s). CV curves reveal the presence of redox peaks for both the samples, demonstrating the pseudocapacitive nature of both CFO and CFO-N. A notable difference in the peak current was observed for both samples at low and high scan rates of 10 mV/s and 100 mV/s respectively, which can be attributed to diffusion-controlled reaction kinetics as well as faster electronic and ionic transport in the electrode/electrolyte interface. CFO-N sample shows well defined anodic and cathodic peaks even after 1000 cycles (Fig. 11c,d), which is attributed to a higher stability than CFO. Hence, it delivers a higher specific capacitance with good cycling stability due to the presence of Fe-N_x_ active sites. Specific capacitance values were calculated from CV curves using the expressions (3) and (4) shown below.

3 $${C}_{sp}=\frac{\int i\,dV}{S\Delta V.m}$$

where $\int i\,dV$ denotes the integral area of CV curve, $\Delta V$ denotes the potential window (V), m is mass of the active material (g), and S is the scan rate (mVs^−1^). The calculated specific capacitance (C_sp_) values are 175.07 Fg^−1^ and 224.67 Fg^−1^ for CFO and CFO-N respectively for third cycle.

4 $${{\rm{C}}}_{sp}=\frac{I\Delta t}{m\Delta V}$$

where ∆V denotes the potential window (V), m denotes the mass of active material (mg), I (A) is the discharge current density and ∆t (s) denotes the discharge time. The specific capacitances from charge-discharge curves are calculated to be 75 Fg^−1^ and 165 Fg^−1^ for CFO and CFO-N respectively. CFO-N exhibits higher C_sp_ and longer discharge time than CFO. Both CV and charge-dicharge curves point towards improved capacitive properties of CFO-N over CFO.

**Figure 12:**
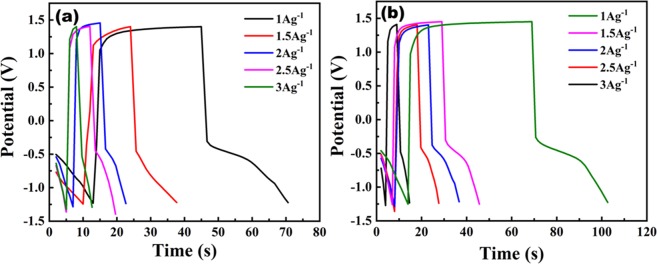
GCD curves of (**a**) CFO (**b**) CFO-N.

The Galvanostatic charge discharge (GCD) studies of CFO and CFO-N are shown in Fig. 12. GCD studies are carried out using chrono-potentiometric analysis at a current density of 1 Ag^−1^, which revealed the capacitive behavior of CFO and CFO-N. The GCD studies are consistent with the CV curves. A non-linear discharging behavior is observed with various current densities. The curvature in charge and discharge profile indicates the capacitive behavior of material, which is influenced by both redox reactions (pseudocapacitor) and electrical double layer (EDLC) response, is similar to reported trends^44–47^. The specific capacitance values were calculated using the expression below (4)

5 $${{\rm{C}}}_{sp}=\frac{I\Delta t}{m\Delta V}$$

CFO-N achieved a high specific capacitance of 160.73 Fg^−1^ at 1 Ag^−1^ with a large potential window of 2.6 V whereas CFO achieved only 109.19 Fg^−1^. A higher potential window implies high energy density of the electrode material. CFO-N exhibits higher C_sp_ and longer charge discharge time as compared to CFO. Both CV and GCD curves point towards improved capacitive properties of CFO-N over CFO. The stability of both materials at various current densities (using GCD plots) are discussed in detail in the supporting information.

**Figure 13:**
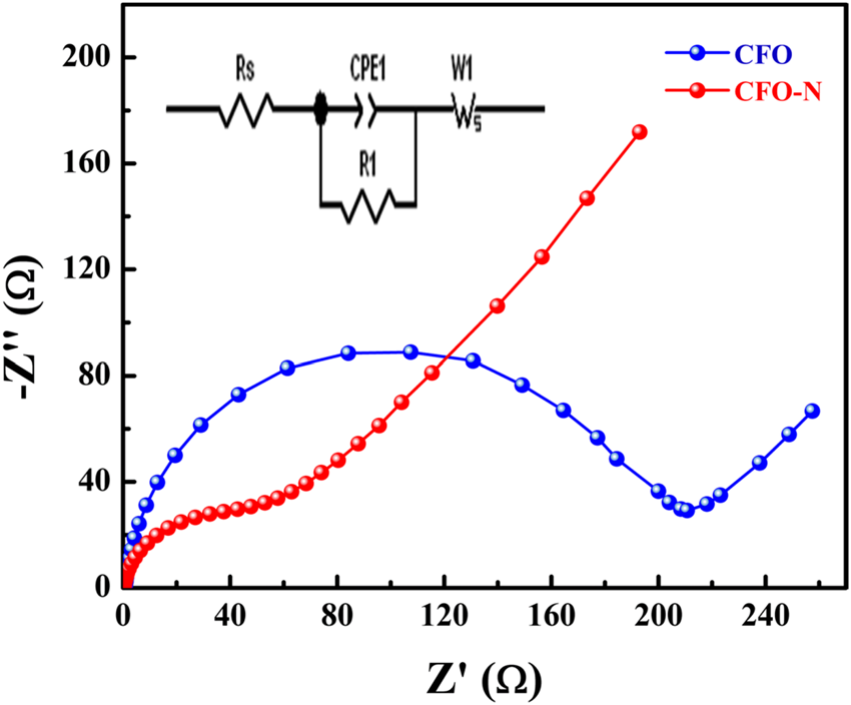
Electrochemical impedance spectra of CFO and CFO-N (inset shows an equivalent circuit).

Electrochemical impedance spectroscopy measurements were carried out for both samples (Fig. 13). From the impedance plot, it is observed that CFO and CFO-N offer negligible series resistance (R_s_) at high frequencies. Both the samples exhibit the same trend exhibiting high-frequency semicircular arc followed by a straight line at lower frequencies. These studies reveal that both are promising candidates for capacitive applications. However, CFO-N is found to be a better candidate than CFO as it offers lower charge transfer resistance. Electrochemical studies revealed better capacitive behavior for CFO-N as compared to CFO. Hence nitrogen doped brownmillerites can be promising materials for energy and environmental applications.

## Conclusion

CFO and CFO-N nanoparticles were synthesized using a chemical route followed by thermoammonolysis. The effect of N incorporation on the bandgap and photocatalytic properties of brownmillerite CFO has been examined. The incorporation of N into CFO significantly decreases the band gap by occupying N 2p energy levels over the O 2p level in the valence band. The electronic band structures of CFO and CFO-N were demonstrated using UPS and UV-vis spectroscopy and the trend was supported by DFT studies. Photodegradation of methylene blue under sunlight revealed superior photocatalytic performance of CFO-N over undoped CFO. Electrochemical studies revealed the feasibility for supercapacitor applications, where CFO-N showed better specific capacitance over CFO. Hence, sunlight-driven photocatalytic degradation of MB and supercapacitor properties expand the scope of utilization of CFO and CFO-N for energy and environmental applications.

## Supplementary information

Supplementary information.
