# Supplementary material for: Nitrogen Incorporated Photoactive Brownmillerite Ca2Fe2O5 for Energy and Environmental Applications
Source: Sci Rep. 2020 Feb 17;10:2713. doi: 10.1038/s41598-020-59454-w (PMC7026084; doi:10.1038/s41598-020-59454-w)
Supplement: Supplementary file 1 — Supplementary information. [file 41598_2020_59454_MOESM1_ESM.doc]

**Nitrogen Incorporated Photoactive Brownmillerite Ca_2_Fe_2_O_5_ for Energy and Environmental Applications**

Durga Sankar Vavilapalli^1^, Soma Banik^2,3^, Raja Gopal Peri^4^, B. Muthuraaman

^4^, M. Muralidhar^5^, M. Murakami^5^, A. Klimkowicz^5^, K. Asokan^6^, M.S. Ramachandra Rao^7^, Shubra Singh ^1*^

*^1^Crystal Growth Centre, Anna University, Chennai-600025, India.*

*^2^Synchrotron Utilization Section, Raja Ramanna Centre for Advanced Technology, Indore-452013, India.*

*^3^Homi Bhabha National Institute, Training School Complex, Anushakti Nagar, Mumbai-400094, India.*

*^4^Department of Energy, University of Madras, Chennai-600025, India*

*^5^Graduate School of Science and Engineering, Shibaura Institute of Technology, 3-7-5 Toyosu, Koto-ku, Tokyo 135-8548, Japan.*

*^6^Materials Science Division, Inter University Accelerator Centre, New Delhi, 110067, India*

*^7^Nano Functional Materials Technology Centre, Department of Physics, Indian Institute of Technology Madras, Chennai-600036, India.*

*^*^Corresponding author’s email:* [*shubra6@gmail.com*](mailto:shubra6@gmail.com)

*
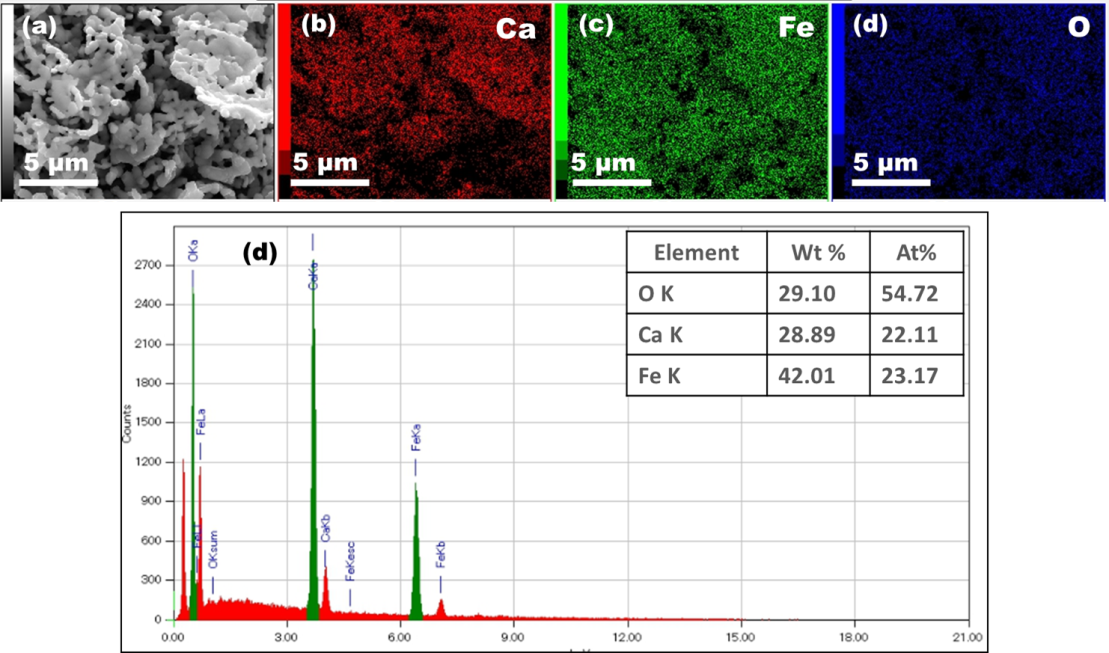
*

Figure S1: SEM image of CFO (b-d) EDX mapping images of CFO sample showing uniform distribution of Ca, Fe and O elements (d) Energy dispersive X-ray (EDX) spectra of CFO (inset weight and atomic percentage of each element )


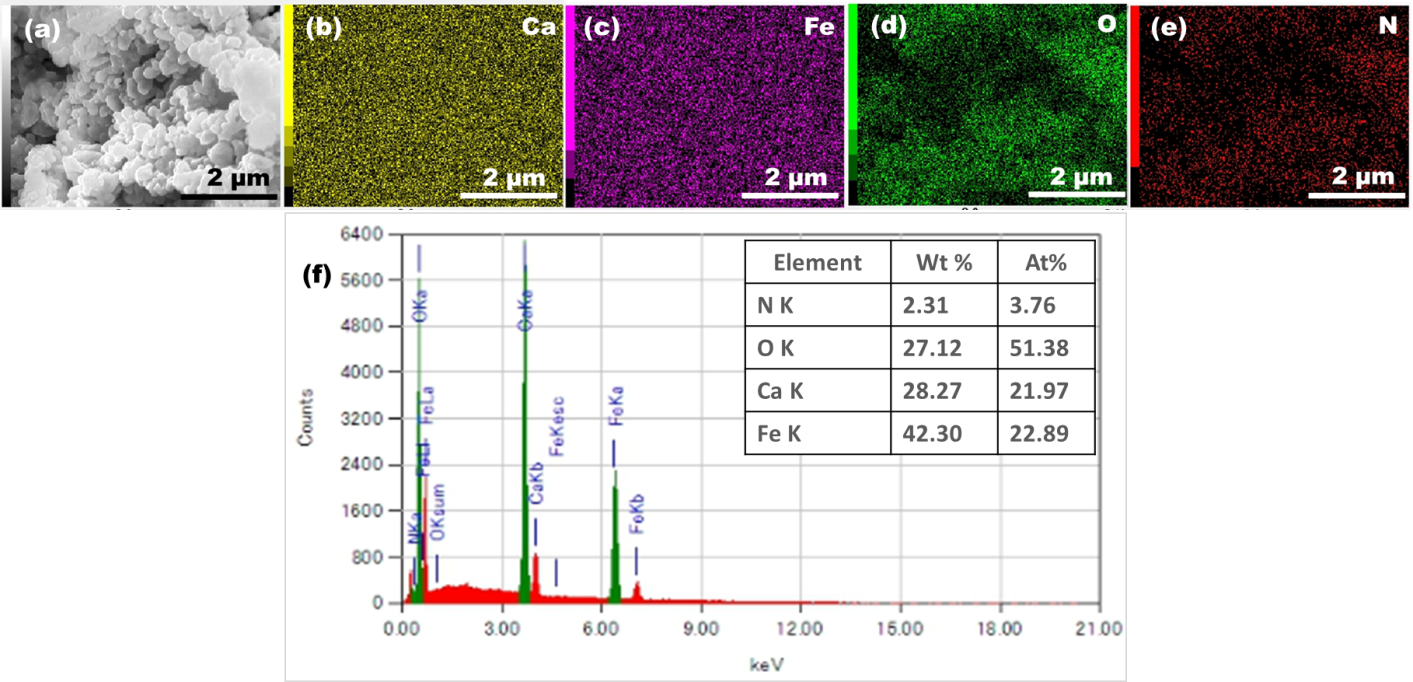


Figure S2: SEM image of CFO-N (b-e) EDX mapping images of CFO-N sample showing uniform distribution of Ca, Fe ,O and N elements (f) Energy dispersive X-ray (EDX) spectra of N-CFO (inset weight and atomic percentage of each elements )


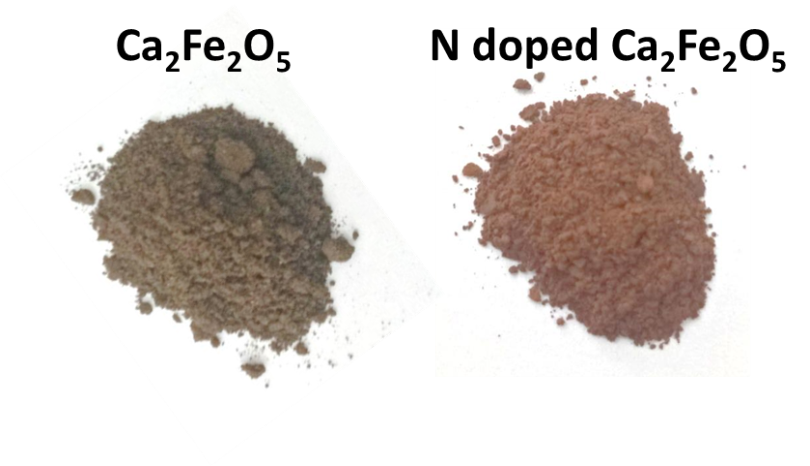


Figure S3. CFO before and after N doping

**Density Functional Theory (DFT) calculations :**

Self-consistent calculations are performed using plane wave basis within the framework of density-functional theory (DFT) [1,2] as implemented within the Quantum ESPRESSO code [3]. We have chosen ultra-soft pseudo-potentials generated using Perdew- Burke-Ernzerhof (PBE) relevant for the generalized gradient approximation (GGA) formalism, to capture the exchange and correlation part of electron-electron interactions [4,5]. In the pseudo-potential description, the electronic configurations (i.e., Ca: 3s^2^ 3p^6^ 4s^2^, Fe: 3d^6^ 4s^2^, O: 2s^2^ 2p^4^ and N: 2s^2^ 2p^3^) are treated explicitly as valence electrons. A plane wave basis set with the kinetic energy cutoff of 140 Ry is used for the expansion of the ground state electronic wave function. Brillouin zone integrations are performed on the Monkhorst-Pack K-point mesh (Monkhorst and Pack, 1976). The grid size used for structural optimization and total energy calculations are 3 × 1 × 3.

In this paper, the [electronic structure calculation](https://www.sciencedirect.com/topics/materials-science/electronic-structure-calculations) is performed by sampling the Brillouin zone with a set of high symmetry k-points [6]. This also enables the calculation of the density of states.

Density functional theory (DFT) [1], in the Kohn-Sham implementation [2] with local density (LDA [7]) or generalized gradient (GGA [8]) approximations for the exchange-correlation (XC) functional, has been successfully applied to deduce structural, electronic, magnetic and other properties of a myriad of condensed matter systems. However, the well-known ‘‘band gap problem’’, in which DFT in LDA/GGA fails to correctly predict the energy gaps between occupied and unoccupied states, is a hindrance to research in fields including semiconductors, optical and photovoltaic materials, and thermoelectrics.

In a typical DFT calculation, the Kohn-Sham gap EKS, i.e., difference between eigen values of lowest unoccupied and highest occupied eigen states, is identified as the bandgap. Typically, EKS underestimates band gaps (EExp) of solids by 30%-100% [9]. The underestimation of EExp by EKS in local and semi-local functionals has been attributed to their inherent lack of derivative discontinuity [10] and delocalization error [11]. It is often claimed that the band gap is an excited-state property and therefore cannot be described by ground state DFT [12].

**References**

1. Hohenberg P, Kohn W. Inhomogeneous Electron Gas. Physical Review 136, B864-B871 (1964).

2. Kohn W, Sham LJ. Self-Consistent Equations Including Exchange and Correlation Effects. Physical Review 140, A1133-A1138 (1965).

3. Giannozzi P, et al. QUANTUM ESPRESSO: a modular and open-source software project for quantum simulations of materials. Journal of Physics: Condensed Matter 21, 395502 (2009).

4. Perdew JP, Burke K, Ernzerhof M. Generalized Gradient Approximation Made Simple. Physical Review Letters 77, 3865-3868 (1996).

5. Perdew JP, Burke K, Wang Y. Generalized gradient approximation for the exchange-correlation hole of a many-electron system. Physical Review B 54, 16533-16539 (1996).

6. Monkhorst HJ, Pack JD. Special points for Brillouin-zone integrations. Physical Review B 13, 5188-5192 (1976).

7. Setyawan W, Curtarolo S. High-throughput electronic band structure calculations: Challenges and tools. Computational Materials Science 49, 299-312 (2010).

8. Langreth DC, Perdew JP. Theory of nonuniform electronic systems. I. Analysis of the gradient approximation and a generalization that works. Physical Review B 21, 5469-5493 (1980).

9. Chan MKY, Ceder G. Efficient Band Gap Prediction for Solids. Physical Review Letters 105, 196403 (2010).

10. Sham LJ, Schlüter M. Density-Functional Theory of the Energy Gap. Physical Review Letters 51, 1888-1891 (1983).

11. Mori-Sánchez P, Cohen AJ, Yang W. Localization and Delocalization Errors in Density Functional Theory and Implications for Band-Gap Prediction. Physical Review Letters 100, 146401 (2008).

12. Godby RW, Schlüter M, Sham LJ. Quasiparticle energies in GaAs and AlAs. Physical Review B 35, 4170-4171 (1987).

**The adsorption curves of MB over different photocatalysts:**


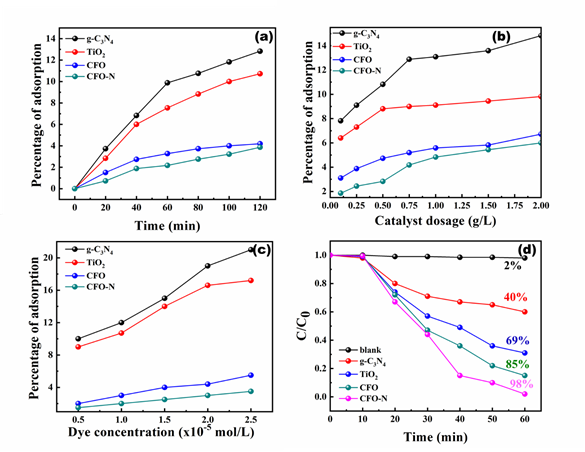


**Figure S4:** Adsorption curves of MB by varying (a) contact time (b) catalyst dosage (c) dye concentration (d) photodegradation performance of CFO and CFO-N over g-C_3_N_4_ and TiO_2_

The adsorption curves of MB using CFO and CFO-N were analyzed over well-known catalysts TiO_2_ and g-C_3_N_4_. Since CFO and CFO-N are visible light active photocatalysts, we investigated its adsorption as well as photodegradation performance over UV-light active catalyst TiO_2_ and visible light active catalyst g-C_3_N_4_. The adsorption features of the catalysts are analyzed by varying the contact time, catalyst dosage and dye concentration.

Initially 1x10^-5^mol/L of MB solution was prepared and 0.5g/L catalyst was loaded to dye solution and sonicated in dark. At every 20 min interval, the dye-catalyst solution was collected and centrifuged and the concentration of MB was measured using UV-Vis absorption spectroscopy. For CFO and CFO-N, an equilibrium in the percentage of adsorption was attained after 40 min. TiO_2_ and g-C_3_N_4_ took longer time to attain equilibrium, as shown in Figure a. The percentage of adsorption is much higher in g-C_3_N_4_ and TiO_2_ compared to CFO and CFO-N. In another study, the contact time was fixed to 60 min and the catalyst dosage was varied, as shown in Figure (b). It was found that CFO, CFO-N and TiO_2_ attained equilibrium at catalyst dosage ~0.5g/L, whereas for g-C_3_N_4_ it was observed at 0.75g/L. The adsorption properties of catalysts were also investigated by varying dye concentration and keeping the contact time, (60 min) and catalyst dosage (0.5g/L) as constants [figure (C)]. Adsorption plots showed similar trend with increase in dye concentration ; the percentage of adsorption increased for all catalysts. The adsorption studies revealed that g-C_3_N_4_ and TiO_2_ have higher percentage of adsorption over CFO and CFO-N.

The photocatalytic degradation performance of g-C_3_N_4_, TiO_2_, CFO and CFO-N were tested under visible light, as shown in figure d. Dye solution, with concentration of 1x10^-5^ mol/L MB was loaded with 0.5g/L catalyst. Upon 50 minutes of light exposure the photodegradation efficiencies of g-C_3_N_4_, TiO_2_, CFO and CFO-N were 40%, 69%, 85% and 98% respectively. CFO-N showed better photodegradation efficiency over other catalysts. Adsorption and photodegradation studies reveal that, the degradation of MB using CFO and CFO-N is brought about by photocatalysis. Adsorption plays a minor role in degradation of MB.

**Stability of CFO and CFO-N for electrochemical energy storage**

The stability of both CFO and CFO-N was studied using cyclic voltammetry (CV) and galvanostatic charge discharge (GCD) methods up to 1000 cycles and corresponding Specific capacitance (C_sp_) values from CV and GCD plots were calculated using the following expressions:

for calculating
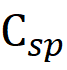
 from CV data
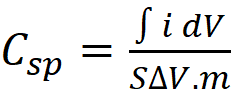
 (1)

for calculating
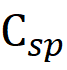
 from GCD data
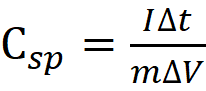
 (2)

where,
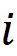
 is the current density,
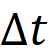
 is the charging–discharging time,
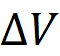
 is the potential range and
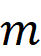
 is the mass of the electroactive material. The calculated
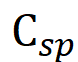
 at various cycles are tabulated in Table1.

**Table 1:** Specific capacitances of CFO and CFO-N calculated from CV and GCD at various cycles.

| **Cycle** | **Specific Capacitance** | | | |
| --- | --- | --- | --- | --- |
|  | **Cyclic Voltammetry** | | **Charge Discharge** | |
|  | **CFO** | **CFO-N** | **CFO** | **CFO-N** |
| 3 | 175.07 | 224.67 | 109.19 | 160.73 |
| 200 | 174.36 | 223.65 | 99.83 | 154.36 |
| 400 | 173.02 | 220.23 | 95.012 | 153.02 |
| 600 | 171.94 | 216.84 | 91.842 | 145.94 |
| 800 | 170.23 | 215.16 | 89.758 | 140.23 |
| 1000 | 168.49 | 214.2 | 85.107 | 138.49 |


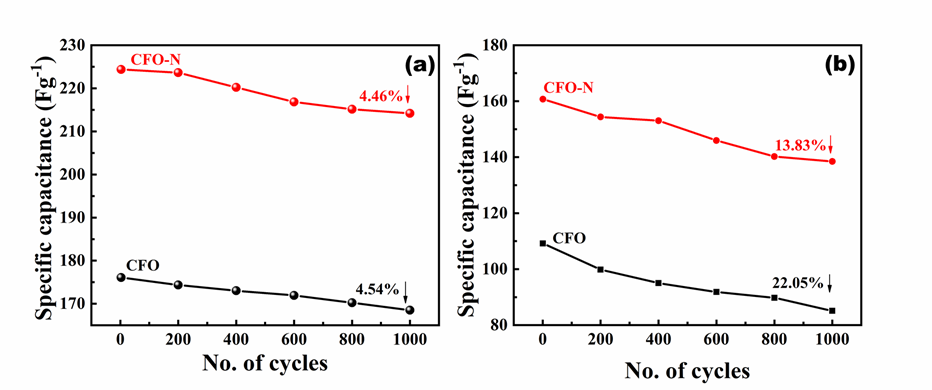


**Figure S5:** Specific capacitances of CFO and CFO-N calculate at various cycles from (a) CV (b) GCD

From cyclic voltammetry data both the materials exhibited a good cycling performance with a small capacitance loss of 4.46 % and 4.54 % after 1000 cycles for CFO and CFO-N respectively, as shown in Figure 2(a) . Both systems (CFO and CFO-N) were analyzed by means of CV at a scan rate of 100 mVs^−1^.

Further, the life cycle of both the compounds, upon 1000 continuous charge discharge cycles, was studied using GCD method at a specific current of 1 Ag^-1^. Figure 2(b) displays the result of the long-term cyclic stability test. The specific capacitance loss is 22.05 % and 13.83 % for CFO and CFO-N respectively. The stability studies imply that, CFO-N is slightly more stable than CFO.

**Tabel S2 :** Specific capacitance of CFO and CFO-N at various current densities.

|  | **Specific Capacitance** | |
| --- | --- | --- |
| **Current density (Ag^-1^)** | **CFO** | **CFO-N** |
| 1 | 109.19 | 160.73 |
| 1.5 | 77.88 | 109.61 |
| 2 | 67.31 | 107.69 |
| 2.5 | 57.69 | 91.34 |
| 3 | 40.38 | 63.46 |
